# Thermophysical Properties of Multifunctional Syntactic Foams Containing Phase Change Microcapsules for Thermal Energy Storage

**DOI:** 10.3390/polym13111790

**Published:** 2021-05-28

**Authors:** Francesco Galvagnini, Andrea Dorigato, Luca Fambri, Giulia Fredi, Alessandro Pegoretti

**Affiliations:** Department of Industrial Engineering and INSTM Research Unit, University of Trento, Via Sommarive 9, 38123 Trento, Italy; andrea.dorigato@unitn.it (A.D.); luca.fambri@unitn.it (L.F.); giulia.fredi@unitn.it (G.F.); alessandro.pegoretti@unitn.it (A.P.)

**Keywords:** syntactic foams, epoxy, glass microspheres, thermal energy storage, phase change materials, thermal properties

## Abstract

Syntactic foams (SFs) combining an epoxy resin and hollow glass microspheres (HGM) feature a unique combination of low density, high mechanical properties, and low thermal conductivity which can be tuned according to specific applications. In this work, the versatility of epoxy/HGM SFs was further expanded by adding a microencapsulated phase change material (PCM) providing thermal energy storage (TES) ability at a phase change temperature of 43 °C. At this aim, fifteen epoxy (HGM/PCM) compositions with a total filler content (HGM + PCM) of up to 40 vol% were prepared and characterized. The experimental results were fitted with statistical models, which resulted in ternary diagrams that visually represented the properties of the ternary systems and simplified trend identification. Dynamic rheological tests showed that the PCM increased the viscosity of the epoxy resin more than HGM due to the smaller average size (20 µm vs. 60 µm) and that the systems containing both HGM and PCM showed lower viscosity than those containing only one filler type, due to the higher packing efficiency of bimodal filler distributions. HGM strongly reduced the gravimetric density and the thermal insulation properties. In fact, the sample with 40 vol% of HGM showed a density of 0.735 g/cm^3^ (−35% than neat epoxy) and a thermal conductivity of 0.12 W/(m∙K) (−40% than neat epoxy). Moreover, the increase in the PCM content increased the specific phase change enthalpy, which was up to 68 J/g for the sample with 40 vol% of PCM, with a consequent improvement in the thermal management ability that was also evidenced by temperature profiling tests in transient heating and cooling regimes. Finally, dynamical mechanical thermal analysis (DMTA) showed that both fillers decreased the storage modulus but generally increased the storage modulus normalized by density (E′/ρ) up to 2440 MPa/(g/cm^3^) at 25 °C with 40 vol% of HGM (+48% than neat epoxy). These results confirmed that the main asset of these ternary multifunctional syntactic foams is their versatility, as the composition can be tuned to reach the property set that best matches the application requirements in terms of TES ability, thermal insulation, and low density.

## 1. Introduction

Syntactic foams (SFs) are an innovative class of materials with tailorable properties and are classifiable both as foams and as composites. Their microstructure, obtained by incorporating hollow rigid particles in a matrix, allows a simultaneous reduction in density and an increase in mechanical properties compared to neat polymers. The resulting materials show higher tensile, higher compressive, and higher impact properties per unit weight than traditional foams, but also higher heat insulation performance, dielectric properties, flame resistance, and sound absorption capacity [1]. This unique combination of properties justifies the intensive research and application of syntactic foams in high-end and weight-sensitive areas, especially in the fields of transportation, aerospace, and marine fields where syntactic foams are employed to produce thermal insulating panels, stiff lightweight cores in composite sandwich structures, deep-sea pipelines, and other diving equipment [2,3,4,5,6,7].

Matrices and particles of SFs can be constituted by a broad range of materials. Matrix materials can be polymeric, ceramic, or metallic. Polymeric matrices are the most diffused due to their low density, low cost, and easy processability while metallic and ceramic foams are employed in niche applications requiring exceptional resistance to high temperatures or harsh environment [8]. Hollow particles can be made of various materials, such as glass, boron carbide, silica, and other ceramics; and with different shapes, such as spherical, cubic, cuboid, and cylindric [4]. The most widely studied syntactic foams are those composed of hollow glass microspheres (HGM) and an epoxy matrix [2,9,10,11,12,13]. In fact, although thermoplastic matrices have attracted much interest due to their recyclability and post-thermoforming potential [2,14,15], the thermomechanical performance of epoxy resins are still unmatched and thus they are the elective materials for high-end applications in aerospace and automotive fields.

The most interesting feature of polymeric SFs containing HGM is their high versatility. The final density and thermomechanical properties can be tailored by varying the properties of the HGM in terms of size, size distribution, volume fraction, surface chemistry and morphology, and shell thickness. For example, the thermal conductivity of polymeric SFs can either increase or decrease with the HGM concentration depending on the ratio between the volume and the wall thickness of hollow spheres [15]. Such remarkable versatility can be further enhanced by adding a third constituent [3]. The property set of epoxy/HGM SFs has been extended by incorporating microfillers and nanofillers such as milled carbon fibers and carbon nanofibers (CNFs), expanded graphene nanoplatelets (xGnPs), nanoclays, and halloysite nanotubes. For example, the incorporation of milled carbon fibers in an epoxy/HGM SF was proven to enhance the fracture toughness [16] and/or to increase the electrical conductivity of the foams, thus rendering these materials useful for electromagnetic interference (EMI) shielding applications [17]. In this sense, SFs are ideal multifunctional materials since their composition can be fine-tuned to obtain an interesting combination of mechanical and functional properties, which are useful when load-bearing and weight-saving requirements must be coupled with additional functionalities.

An interesting functional property that could be added to SFs is the thermal energy storage (TES) ability, i.e., the capacity of temporarily storing heat that can be released when and where needed [18,19,20,21,22]. TES technologies are used for thermal management in a wide variety of applications, including transportation and refrigeration chain, buildings, food containers, and smart textiles [18,23,24]. The most diffused TES technique in the low-mid temperature range (0−100 °C) harnesses the melting–freezing phase change of organic phase change materials (PCMs) such as paraffin waxes, which store and release a considerable amount of latent heat (up to 250 J/g) at a nearly constant temperature [25,26,27]. In order to avoid leakage and loss of material above the melting temperature, PCMs are often encapsulated in stable micro-shells and the resulting PCM microcapsules are non-toxic and easy to be incorporated in a polymer matrix [28,29,30]. Our group has recently investigated the mechanical and TES properties of polymer composites containing such PCM microcapsules and reinforced it with continuous or discontinuous fibers [20,21,31,32,33,34,35,36], which resulted in multifunctional structural TES composites useful for applications where weight saving, and thermal management are equally important. Similarly, adding PCM microcapsules to polymeric SFs could result in multifunctional materials with tunable thermomechanical properties, to be applied in the automotive and aerospace fields, where low density, thermal management capability, and excellent compressive and flexural properties are often required simultaneously. Despite the potentialities of such epoxy/HGM/PCM ternary systems, no papers can be found in the open scientific literature about syntactic foams containing PCMs to the best of the authors’ knowledge.

This work, therefore, aims to expand the property set of epoxy/HGM syntactic foams via the introduction of a microencapsulated PCM possessing a phase change temperature of 43 °C to produce a material with an interesting combination of highly specific mechanical properties and thermal management capability. To realize this aim, fifteen epoxy/HGM/PCM composites were prepared with different HGM-to-PCM ratios, with a maximum total filler content of 40 vol% and the compositions were chosen by using a design of experiment (DOE) approach. The subsequent characterization focused on the rheological, microstructural, and thermal properties of the prepared systems. First, the processability was assessed by evaluating the rheological properties of uncured epoxy/HGM/PCM mixtures as a function of the filler type and concentration. Then, the microstructure and the density of the resulting foams were investigated by utilizing scanning electron microscopy (SEM) and pycnometer density, respectively. The TES properties were studied by differential scanning calorimetry (DSC) and by monitoring the inner temperature of the samples during heating/cooling ramps. Finally, the thermal conductivity was investigated by light flash analysis (LFA), while their viscoelastic properties were evaluated at different temperatures by utilizing dynamic-mechanical thermal analysis (DMTA).

## 2. Materials and Methods

### 2.1. Materials

The epoxy base EC 157 (density = 1.15 g/cm^3^, viscosity at 25 °C = 600–800 mPa∙s) and the amine hardener W342 (density = 0.95 g/cm^3^, viscosity at 25 °C = 30–70 mPa∙s) were kindly provided by Elantas Europe Srl. (Collecchio, Italy). The K15 hollow glass microspheres (HGM) were provided by 3M Italia Srl. (Pioltello, Italy). As reported on the technical datasheet of the producer, they were made of soda-lime-borosilicate glass and had a density of 0.15 g/cm^3^, a mean particle size (D50) equal to 60 μm, a thermal conductivity of 0.055 W/(m∙K), and a crush strength (90% survival) of 2.07 MPa. The PCM used in this work was an encapsulated paraffin MPCM43D, provided by Microtek laboratories Inc. (Dayton, USA), possessing a density of 0.9 g/cm^3^ and a mean size of 17–22 µm. The melamine–formaldehyde shell, constituting the 15 wt% of the total mass, guarantees the thermal stability up to 250 °C. The paraffinic core had a melting temperature of 43 °C and a melting enthalpy of 190–200 J/g. All materials were used as received.

### 2.2. Sample Preparation

**Figure 1:**
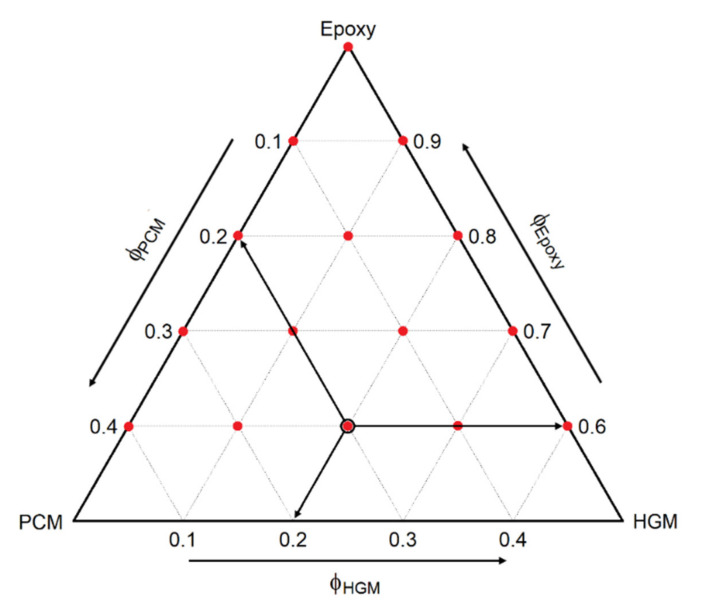
Graphical representation of the prepared compositions (red dots) on the ternary diagram. The black bordered dot refers to the EPG-20.20 foam, possessing a PCM concentration of 20 vol% and a HGM concentration of 20 vol%.

Epoxy/PCM/HGM composites were prepared by mixing the epoxy base with PCM and HGM at 100 rpm for one minute through a Dispermat F1 mechanical mixer provided by VMA-Getzmann GmbH (Reichshof, Germany). The mixture was then vacuum-degassed for 5 min. After the addition of the hardener, the resulting compounds were further mixed for 1 min and vacuum-degassed for 5 min. The mixtures were then casted in silicon molds with different geometry and cured for 24 h at room temperature and 6 h at 80 °C. This procedure was adopted to prepare 15 compositions, which are listed in Table 1. Samples were labeled as EPG-x.y, where x represents the concentration (vol %) of PCM and y represents that of HGM. These formulations were selected by using a specific mixture design performed with the software RStudio v.1.4.1103 (RStudio Inc., Boston, MA, USA). The result of such design is represented in Figure 1, which shows the chosen compositions with red dots on a ternary phase diagram. In this paper, the results of the main physical properties of these foams were graphically reported via ternary diagrams similar to that reported in Figure 1.

### 2.3. Experimental Techniques

#### 2.3.1. Rheological and Morphological Properties

The rheological properties play an important role in determining the processing window of thermosetting materials. Hence, a DHR-2 rheometer (TA instrument, New Castle, DE, USA) was used to perform two different analyses on the uncured mixtures by adopting a plate–plate configuration with a gap distance of 1 mm. For both tests, one specimen was tested for each composition. The first test consisted in analyzing the shear rate sensitivity between 0.1 and 1 s^−1^ of 7 compositions, i.e., EPG-0.0, EPG-0.20, EPG-0.40, EPG-10.10, EPG-20.0, EPG-20.20, and EPG-40.0 at a constant temperature of 30 °C. The second test analyzed the dynamic rheological behavior of the mixtures at 70 °C, 80 °C, 90 °C, and 110 °C applying a maximum shear stress of 1000 Pa at a constant frequency of 1 Hz. This test was performed on 4 compositions, i.e., EPG-0.0, EPG-0.40, EPG-40.0, and EPG-20.20 and allowed determination of the gel time (t_gel_), which is defined as the time at which the curve of the shear storage modulus (G′) intersects with that of the shear loss modulus (G″). These results allowed the determination of the activation energy of crosslinking (E_a_) via the Arrhenius approach, as is reported in Equation (1):

(1) $$E_{a}=R\left(\frac{d\left({\ln\;t}_{\mathrm{gel}}\right)}{d(1/T)}\right)$$

where R is the universal gas constant equal to 8.314 J/(mol∙K) and T is the testing temperature (in K).

The microstructure of syntactic foams was investigated by analyzing the fracture surface of the prepared (cured) samples with a Zeiss Supra 40 (Carl Zeiss AG, Oberkochen, Germany) scanning electron microscope (SEM) operating at an accelerating voltage of 3.5 kV after Pt-Pd sputtering.

Density measurements were performed for all the compositions on small samples of approximately 3g with a Micromeritics AccuPyc 1330TC (Micromeritics Instrument Corp., Norcross, GA, USA) helium pycnometer operating at 23.0 °C and equipped with a testing chamber of 1 cm^3^. One specimen was tested 30 times for each composition and the average of the 30 measurements was presented as the result.

#### 2.3.2. Thermal Properties

Differential scanning calorimetry (DSC) was performed with a Mettler DSC30 instrument (Mettler Toledo LLC, Columbus, OH, USA) on all the prepared foams and on the neat PCM. Specimens of approximately 40 mg were sealed in 160-µl aluminum crucibles and subjected to a cycle of three scans (heating, cooling, and heating) between 10 °C and 160 °C at a heating/cooling rate of +10 °C/min under a nitrogen flow of 100 mL/min. One specimen was tested per composition. These tests allowed the determination of the melting and crystallization temperature of the PCM (T_m1_, T_c_, and T_m2_), the specific melting and crystallization enthalpy of the PCM (ΔH_m1_, ΔH_c_, and ΔH_m2_,), and the glass transition temperature of the epoxy matrix (T_g_). Furthermore, the specific heat (c_p30_) at 30 °C was calculated from the DSC thermograms in the first heating scan by dividing the specific heat flow by the heating rate at 30 °C. Although this method does not comply with ASTM E-1269 standard, the obtained c_p_ values were used to determine, at least from a qualitative point of view, the thermal conductivity of the prepared foams according to Equation (2).

The TES capability of the prepared samples was also investigated on a larger scale by testing the cylindrical specimens (diameter 20 mm, height 35 mm) of some selected compositions, i.e., EPG-0.0, EPG-0.20, EPG-0.40, EPG-10.10, EPG-20.0, EPG-20.20, and EPG-40.0. A blind hole with a diameter of 2 mm and a depth of 20 mm was machined on one base of the specimens to allow for the insertion of a type-K thermocouple connected to a recording system. These specimens were subjected to two different thermal ramps, i.e., from 25 °C to 60 °C and from 60 to 25 °C as they were placed in an oven (60 °C) for 40 min and then pulled out and left for cooling at 25 °C while their inner temperature was recorded at 1 point per second during the thermal transients. In this way, it was possible to obtain the time required by each sample to reach the temperature of 55 °C (t_26–55_) in the heating ramp and the temperature of 26 °C (t_55–26_) in the cooling ramp.

The thermal diffusivity and conductivity of the prepared foams were determined by using a light flash analyzer LFA 467 (Netzsch Holding, Selb, Germany). Tests were performed at 30 °C on seven compositions, i.e., EPG-0.0, EPG-0.20, EPG-0.40, EPG-10.10, EPG-20.0, EPG-20.20, and EPG-40.0. One square specimen (12.7 × 12.7 × 4 mm^3^) for each composition was cut from a cast plate and coated with graphitic spray and three pulses were performed for each sample. These tests allowed the direct measurement of the thermal diffusivity (α). Then, the thermal conductivity at 30 °C (λ_30°C_) was calculated by using Equation (2):

(2) $$\lambda_{30\;{}^{\circ}C}=\alpha \cdot \rho \cdot c_{p30}$$

where ρ is the pycnometer density (at 23.0 °C) and c_p30_ is the specific heat calculated from the first scan of the DSC at 30 °C.

#### 2.3.3. Dynamic-Mechanical Properties

Dynamic mechanical thermal analysis (DMTA) was performed with a TA Q800DMA analyzer (TA Instruments, New Castle, DE, USA) on eight compositions, i.e., EPG-0.0, EPG-0.10, EPG-0.30, EPG-0.40, EPG-10.30, EPG-30.0, EPG-30.10, and EPG-40.0. One rectangular specimen (10 × 4 × 40 mm^3^) for each composition was tested in the single cantilever bending mode by setting a distance of 17.5 mm between grips. Tests were conducted from 0 °C to 150 °C at 3 °C/min, while the strain amplitude and frequency were set to 0.05 % and 1 Hz, respectively. These tests permitted the obtaining of the trends of the storage modulus (E′) and loss tangent (tanδ) as a function of temperature. The values of the storage modulus E′ at 25 °C, 60 °C, and 130 °C were used to elaborate three different ternary plots to better evaluate the E′ trend. These values of E′ were divided by the pycnometer density measured at 23 °C to obtain the specific storage modulus (E′/ρ). Since there was no equipment capable of measuring the density of materials at temperatures other than 23 °C in our laboratory, the pycnometer density at 23 °C was used to normalize the values of E′ acquired at higher temperature.

#### 2.3.4. Design of Experiment (DOE) and Statistical Analysis of the Experimental Data

The analysis of the properties of a ternary system can be a very long process, due to the broad variety of possible compositions. Hence, a statistical approach was implemented in this paper to define the mixture design and represent the obtained results by using the RStudio v.1.4.1103 software (RStudio, Inc., Boston, USA). The “mixexp” package was used for the mixture model part and resulted in 15 selected compositions (see Figure 1 and Table 1). Then, the experimental results were fitted with the “lm” function by a quadratic linear model called “Scheffé quadratic model” [36] as reported in Equation (3)

(3) $$y=\sum_{i=1}^{q}\beta_{i}x_{i}+\sum_{i=1}^{q-1}\sum_{j=i+1}^{q}\beta_{\mathrm{ij}}x_{i}x_{j}+\epsilon$$

where y is the response variable, x_i_ and x_j_ denotes the binary mixture compositions, β_i_ represents the expected response at the vertex, and β_ij_ are the coefficients indicating the amount of quadratic curvature along the edge of the simplex region [37]. After a first fit, the most significant components of the model (x_i_, x_j_) were evaluated through the analysis of variance (ANOVA), while all non-significant components and combinations of components were removed from the model and a new fit with the corrected model was performed. This procedure was repeated until only the statistically significant combinations of components remained. At this juncture, the model can be considered statistically correct and used to represent the analyzed data. The function “ModelPlot” was used to plot the ternary models and the resulting R^2^_adj_ of the fitting model was noted on each plot. This method has been adopted to elaborate all the ternary plots reported in this work.

## 3. Results

### 3.1. Rheological and Morphological Properties

**Figure 2:**
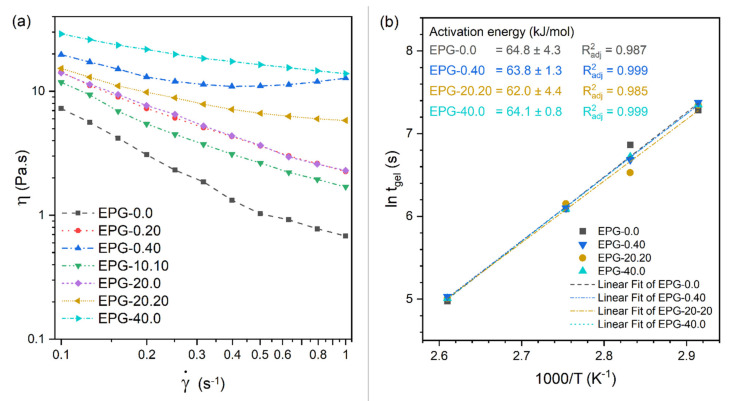
Results of dynamic rheological tests on uncured resins. (**a**) Viscosity (η) as a function of the shear rate ($\dot{\gamma }$) of some selected compositions (T = 30 °C). (**b**) Gel time (t_gel_) as a function of the curing temperature (1000/T) of some selected compositions. Experimental data (symbols) were fitted with Equation (1) (dashed lines) to obtain the activation energy of the crosslinking process.

The processability window of a thermosetting system can be effectively investigated by measuring its rheological properties in the uncured state following the mixing step. The results of the dynamic rheological tests performed on the prepared mixtures are illustrated in Figure 2a,b. The viscosity (η) decreases by increasing the applied shear rate ($\dot{\gamma }$) for all the investigated compositions (Figure 2a). This behavior was expected since the system is still in the liquid state and the very beginning of the curing process is not yet detectable [38]. Conversely, the introduction of solid particles (PCM and/or HGM) determines a significant increase in η, which is especially evident at higher values of $\dot{\gamma }$ and this aspect could reduce the workability of the system. For example, the value of η at 0.2 s^−1^ for the sample EPG-10.10 is 5.5 Pa∙s (+77% compared to EPG-0.0 foam) and for the sample EPG-40.0 is 21.8 Pa∙s (+603% compared to EPG-0.0 sample).

The effect of the type and concentration of solid particles on the viscosity of the liquid resin deserves a deeper insight. When considering samples containing only one particle type (PCM or HGM), the particle type has no effects on viscosity up to a content of 20 vol% and, in fact, the samples EPG-0.20 and EPG-20.0 have very similar values of viscosity over the whole range of shear rate. On the other hand, the particle type does influence the rheological properties at higher filler concentrations, with the PCM increasing the viscosity more than HGM, especially at low shear rates. For example, the viscosity of the sample EPG-0.40 containing 40 vol% of PCM has a viscosity of 13.1 Pa∙s at 0.2 s^−1^, which is approximately 66 % higher than that of the sample EPG-40.0 containing 40 vol% of HGM. This is related to the average particle size. Since PCM has a smaller particle size than HGM (approximately 20 µm vs. 60 µm), the matrix-filler contact area is larger and therefore the tendency to form a percolative network increases, thus leading a more severe increase in viscosity [39].

Considering the samples containing both particle types, EPG-10.10 and EPG-20.20, they have a lower viscosity than those containing a single particle type at the same filler concentration. This is, again, due to the different particle size which influences the maximum theoretical particle volume fraction reached with random close packing configuration and, in turn, the viscosity. The maximum theoretical volume fraction for close-packed spherical particles is 64 vol% when the size distribution is unimodal. However, this value can increase for bimodal or multimodal distributions because fine particles can settle within the interstices of coarser particles [38]. This phenomenon, widely described in the literature, helps to explain the lower viscosity of samples containing both PCM and HGM. In fact, the higher the maximum theoretical packing density, the lower the viscosity at a given concentration [39,40,41,42,43].

Rheological tests carried out on the uncured mixtures at constant shear rate and at four different temperatures (from 70 °C to 110 °C) provides information on the gel time (t_gel_) and allows for the calculation of the activation energy of the crosslinking process for the different compositions (Equation (1)). The results of these tests are illustrated in Figure 2b, which reports the natural logarithm of t_gel_ as a function of 1000/T and the values of activation energy of crosslinking determined from the slope of the linear regressions. Interestingly, the activation energy is not significantly different in the four analyzed compositions, as is found in previous studies on epoxy/HGM systems [44]. This suggests that the inclusion of PCM and/or HGM does not influence the crosslinking mechanism of the epoxy resin and does not narrow the processing window.

**Figure 3:**
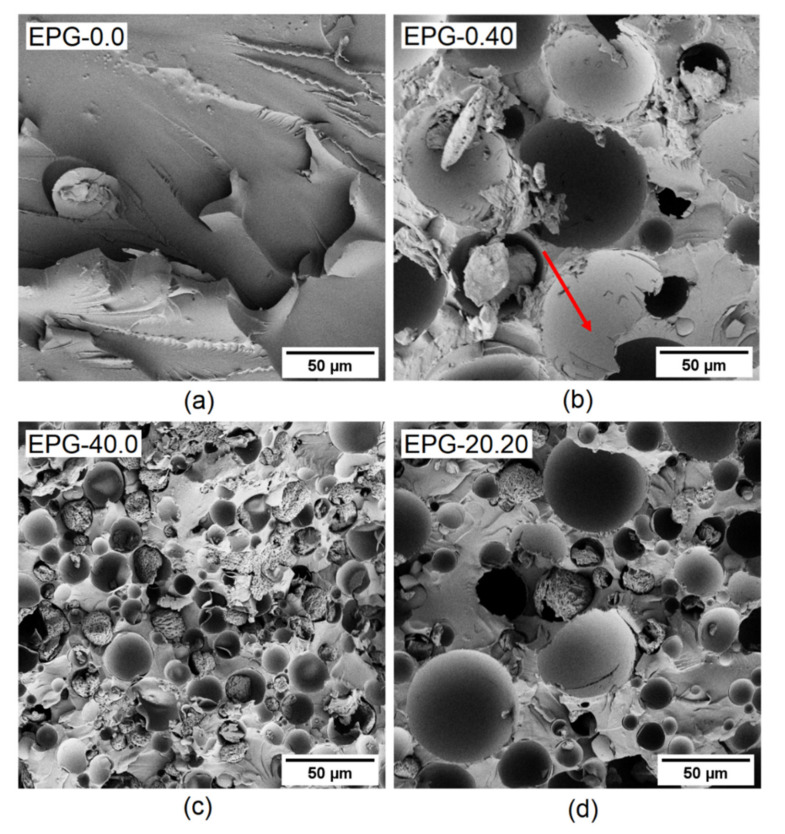
SEM micrographs of the fracture surface of some selected compositions: (**a**) EPG-0.0, (**b**) EPG-0.40, (**c**) EPG-40.0, and (**d**) EPG-20.20 (the arrow indicates some cracks on the HGM shell fragments).

The microstructure of the cured samples was analyzed by utilizing SEM and Figure 3a–d shows the SEM micrographs of the fracture surface of some selected compositions (EPG-0.0, EPG-0.40, EPG-40.0, and EPG-20.20). All samples exhibit a brittle fracture, as evidenced by the flat fracture surface. HGM (Figure 3b) are homogeneously dispersed within the epoxy matrix and with a good interfacial adhesion and this is evidenced by the absence of gaps between the outer shell surface and the matrix. HGM show a brittle behavior themselves and this is observable from the cracks on some shell fragments (as shown by the arrow on Figure 3b). Moreover, the fact that most HGM are empty and with a smooth inner side suggests that the breakage occurred during the fracture process and not during the mixing phase, which confirms that the adopted processing parameters are mild enough to preserve their integrity.

Figure 3c shows the microstructure of the sample EPG-40.0, containing 40 vol% of PCM. The PCM is constituted by core-shell spherical microcapsules with an average diameter of approximately 20 μm which is observed in previous literature [38] and in good agreement with the producer’s datasheet. The adhesion between the outer PCM shell and the epoxy matrix is not optimal and this is better observed on higher-magnification micrographs (not reported for brevity). However, the fact that most PCM microcapsules are broken suggests that the fracture propagates across the microcapsules and not at the interface with the epoxy matrix [45,46]. In any case, the low interfacial adhesion could represent a drawback of the proposed system and will be addressed in future research. Moreover, most PCM capsules still show their rough and irregular paraffinic core, while the core of the smooth empty capsules has probably remained on the other side of the fracture surface. This suggests that the integrity of the PCM is preserved during processing, thereby excluding any possibility for paraffinic core to leak out of the composite during service. A comparison between Figure 3b,c evidences the size difference between PCM and HGM, and this is confirmed from the micrograph of the sample EPG-20.20 (Figure 3d), which also evidences the relative distribution of the fillers. In fact, the smaller PCM capsules are located in the interstices of the bigger HGM in good agreement with the results of the rheological measurements (see Figure 2a).

**Figure 4:**
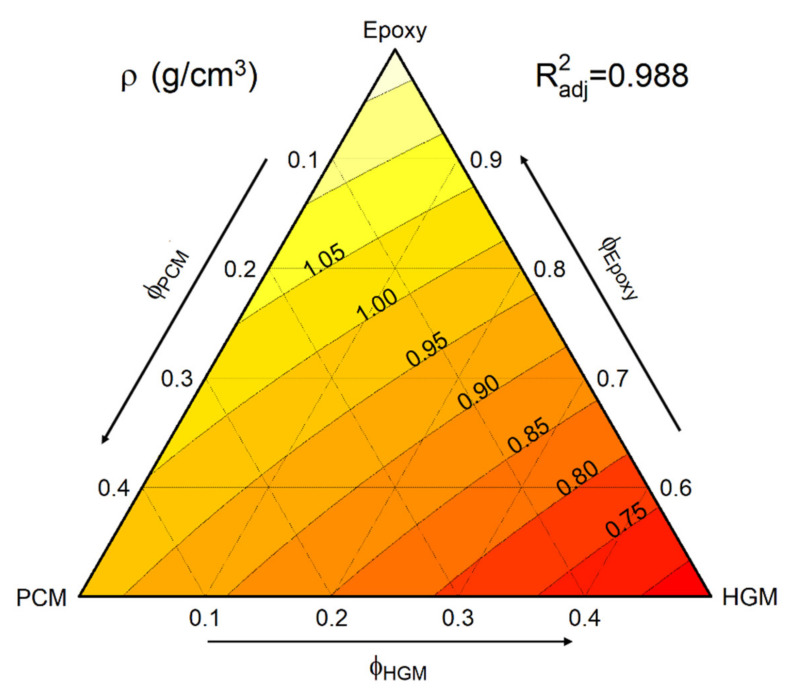
Fit-model of the pycnometer density of the prepared syntactic foams.

One of the most important and interesting aspects of syntactic foams is their capability to reach a very low density while keeping high stiffness and strength. Therefore, the density of the prepared samples was measured via helium pycnometry and the obtained values were fitted with a linear statistical model (as explained in Section 2.3.4) to obtain the ternary diagram reported in Figure 4. The bulk density of neat and cured epoxy is 1.137 g/cm^3^. Since the bulk density of HGM (0.150 g/cm^3^) is lower than that of PCM (0.955 g/cm^3^), it is unsurprising that the density decreases more due to HGM than to PCM. For instance, the density of the sample EPG-20.20 is 0.872 g/cm^3^ (−23 % than neat epoxy), while that of EPG-0.40, containing only HGM but having the same total filler amount, is 0.735 g/cm^3^ (−35 % than neat epoxy). The experimental values of density, thanks to the application of the linear model, can also be applied to evaluate the density for the intermediate compositions. The results of the linear model, obtained with a high R^2^_adj_ value (0.988), are in good agreement with the mixture rule, which suggests that most of the HGM and PCM capsules survived the processing step.

### 3.2. Thermal Properties

**Figure 5:**
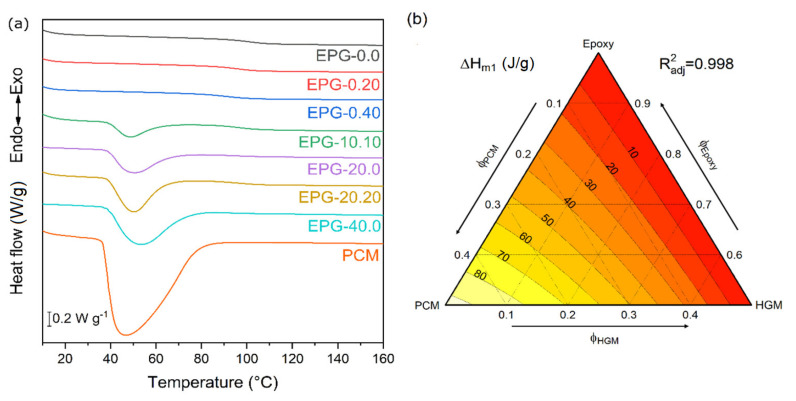
(**a**) DSC thermograms of the neat PCM and of the prepared foams (first heating scan); (**b**) fit-model of the melting enthalpy values (first heating scan) of the prepared foams.

The addition of PCM to epoxy/HGM syntactic foams enriches them with TES properties, i.e., the capability of absorbing and releasing heat in a specific temperature range. Among the techniques to measure TES properties, DSC is one of the most widely used because it requires a small amount of sample and allows the measuring of latent heat stored and released by evaluating the total phase change enthalpy. In this work, DSC was employed to measure not only the melting/crystallization enthalpy of the PCM (ΔH_m1_, ΔH_c_) and the corresponding phase change temperatures (T_m1_, T_c_) but also the glass transition temperature of the epoxy matrix during the first heating scan (T_g1_) and the specific heat of the foams at 30 °C (c_p30_). All these results were collected by performing heating/cooling/heating DSC scans, but only the data of the first heating scan are presented in Figure 5a,b while Table 2 also shows the results of the cooling scan.

Figure 5a shows the DSC thermograms of neat PCM and of some selected compositions. Neat PCM shows a prominent endothermic peak between 35 °C and 60 °C, associated to the melting of the paraffinic core, as is already observed in previous works on the same PCM [33,47]. This signal is also observable in all PCM-containing samples in the same temperature range. The related peak temperatures and enthalpy values are reported in Table 2 as T_m1_ and ΔH_m1_, respectively. The values of T_m1_ slightly increases with PCM concentration, which is probably due to an increased thermal inertia, as is already observed in previous works on the same PCM [38]. In any case, the temperature interval in which the heat is exchanged is very similar for all the investigated samples. It can be noticed that T_c_ is generally lower than T_m1_. This phenomenon, probably due to thermal inertia, is already observable for neat microcapsules for which T_m1_ and T_c_ are 45.9 °C and 27.9 °C, respectively, and is even more evident on all the other samples for which the thermal conductivity is lower. In fact, while T_m1_ varies between 47 °C and 53 °C, T_c_ is spans between 19 °C and 24 °C.

Moreover, the values of ΔH_m1_ increases with the PCM content and are nearly proportional to the PCM weight fraction. The values of ΔH_c_ are very close to those of ΔH_m1_, which highlights the reversibility of the process. The melting enthalpy can be also graphically evaluated from Figure 5b, which shows the results of the fitting of the experimental enthalpy data with the linear ternary model (as reported in Section 2.3.4) obtained with a R^2^_adj_ value of 0.998. Some interesting compositions can be EPG-20.20 and EPG-30.20 (the latter can be extrapolated from the model), which capable of storing and releasing approximately 45 J/g and 68 J/g of thermal energy, respectively.

DSC tests also allowed the determination of T_g1_ and T_g2_ of the epoxy phase and the c_p30_ of the samples, which are all reported in Table 2. The T_g1_ of the neat epoxy sample is approximately 92 °C and does not follow a specific trend with the filler concentration, which confirms that the fillers do not substantially affect the curing process, as shown by rheological tests. However, it is quite difficult to precisely locate the inflection point in DSC thermograms since the signal is less intense at higher filler loadings. The T_g1_ can be better evaluated with DMTA techniques (see Section 3.3). For the values of c_p30_ reported in Table 2, they are mainly affected by the incorporation of PCM. Even though these values have not been measured by following the ASTM E-1269 standard, they are comparable with those found in the literature [10,48] and were thus used to calculate the thermal conductivity.

**Figure 6:**
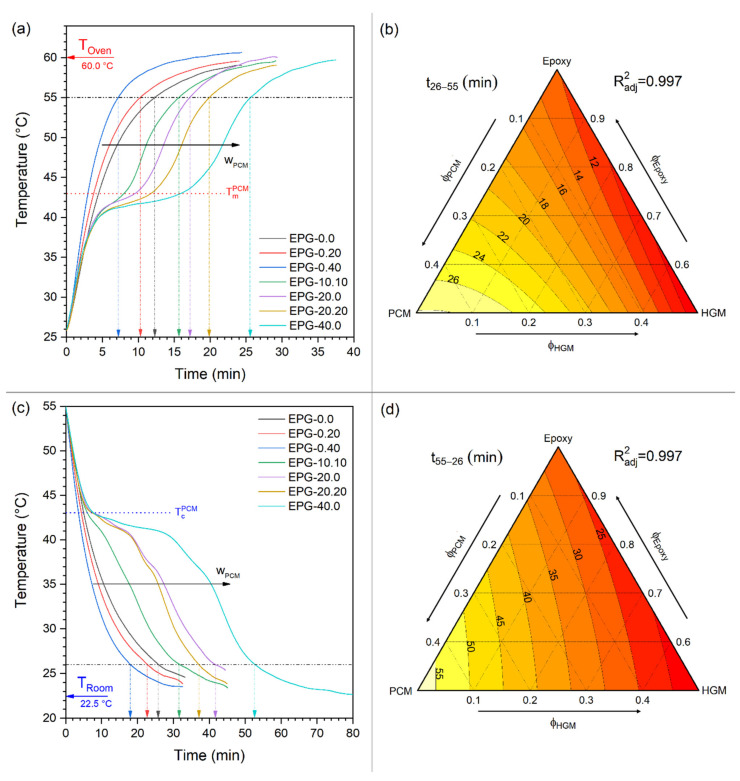
(**a**) Temperature evolution of the prepared syntactic foams during the heating stage, (**b**) fit-model of t_26–55_ values, (**c**) temperature evolution of the prepared syntactic foams during the cooling stage, and (**d**) fit-model of t_55–26_ values.

In order to investigate the TES properties of the prepared samples on a larger scale, a temperature profiling test was carried out during the heating and cooling mode. In this test, cylindric samples with a volume of approximately 10 cm^3^ were first heated in an oven at 60 °C and then left cooling at 25 °C while their inner temperatures were measured with a K-type thermocouple. The results of these tests are reported in Figure 6a–d. Figure 6a,c shows the temperature profiles during the heating and cooling transients, respectively. These profiles clearly highlight the influence of PCM since the samples containing PCM show a plateau-like region at the PCM phase change temperature (43 °C), which is evident especially at higher PCM concentrations. This behavior, already observed in our previous work [20], delays the heating (Figure 6a) and the cooling (Figure 6c) process and increases t_26–55_ and t_55–26_, i.e., the time required for the specimens to reach the surrounding temperature during the heating and cooling transients. For instance, the sample EPG-20.20 takes 19.8 min to reach 55 °C in the heating test (+63% than neat epoxy), while the sample EPG-40-0 takes 25.6 min to reach the same temperature (+111%). These results suggest that the samples with higher PCM loadings are promising for thermal management applications.

The values of t_26–55_ and t_55–26_ were fitted with the linear model (R^2^_adj_ = 0.997) and the results of the fitting (Figure 6b,d) show the effect of the PCM in decreasing the heating and cooling rates. On the other hand, the influence of HGM is unexpected. An increasing HGM content seems to decrease both t_26–55_ and t_55–26_, i.e., to accelerate the heating and cooling processes. The fit-model correctly interprets the experimental data since the temperature profiles of the samples containing only HGM are indeed steeper than that of the neat epoxy. This seems to suggest that HGM favors the thermal exchange, while the opposite is more probably true, given their low declared thermal conductivity. These effects can be due to an incorrect positioning of the thermocouple and can therefore be considered part of the experimental error. In fact, this semi-quantitative test was carried out to highlight the TES effects of PCM and not to quantitatively evaluate the thermal conductivity or diffusivity of the samples.

**Figure 7:**
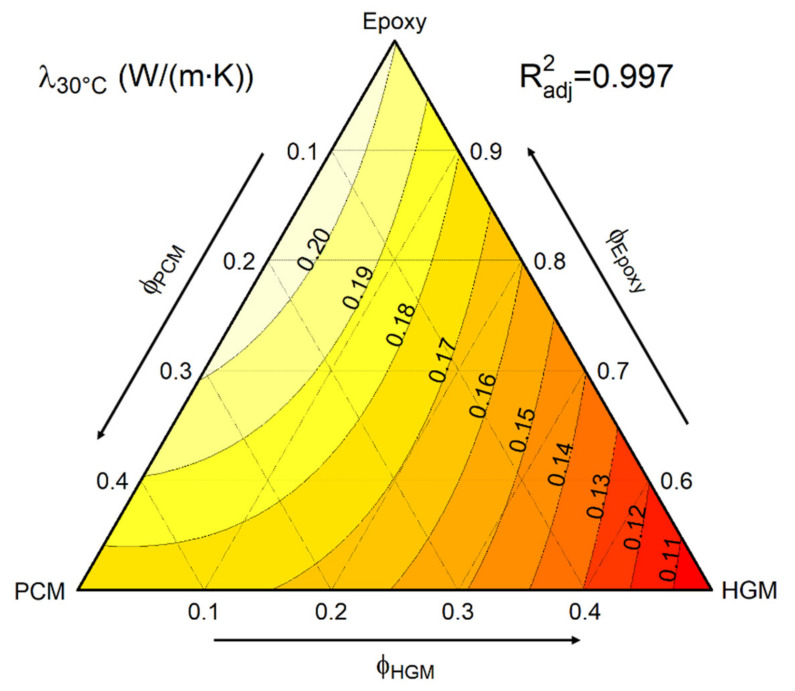
Fit-model of thermal conductivity of the prepared foams at 30 °C.

Conversely, a quantitative evaluation of the thermal conductivity and diffusivity of the prepared foams was performed via light flash analysis (LFA), which confirmed the thermal insulation effect of HGM. The instrument returned the values of thermal diffusivity at 30 °C when used with the value of c_p30_ at 30 °C (Table 2) and the pycnometer density (Figure 4) to calculate the thermal conductivity (λ_30°C_) via Equation (2). The values of thermal conductivity were then fitted with a ternary model, resulting in the plot of Figure 7.

As expected, an increase in HGM content significantly decreases the thermal conductivity, which is in agreement with the results found in previous literature [49]. On the other hand, the PCM promotes only a modest decrease in thermal conductivity. For example, the value of λ_30°C_ for the sample EPG-40.0 is only 5% lower than that of neat epoxy, while for EPG-0.40 the reduction in λ_30°C_ is close to 40% (from 0.20 to 0.12 W/(m∙K)). Therefore, the optimal composition must be chosen according to the intended application of the foam in order to maximize the TES properties or the thermal insulation capability. Maximizing TES capability could be important when the aim is to manage and smooth temperature peaks of limited duration, while a low value of thermal conductivity could be preferred when the temperature conditions are stationary. Intermediate compositions, such as EPG-20.20 or EPG-30.20, could be a good compromise to match both requirements.

### 3.3. Dynamic-Mechanical Thermal Analysis (DMTA)

**Figure 8:**
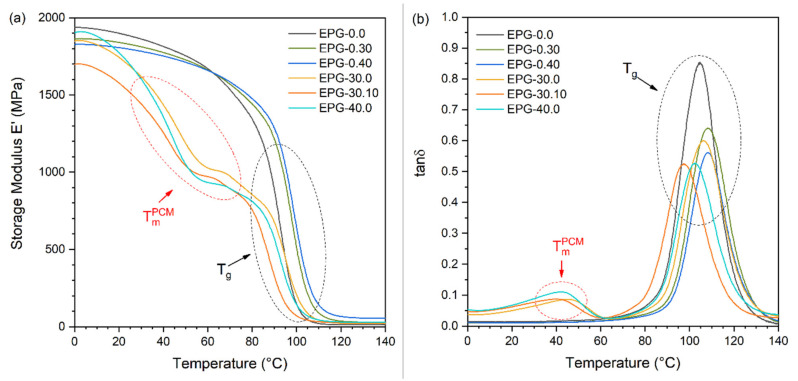
DMTA thermograms of the prepared foams. (**a**) Storage modulus E′ and (**b**) tanδ as a function of temperature.

**Figure 9:**
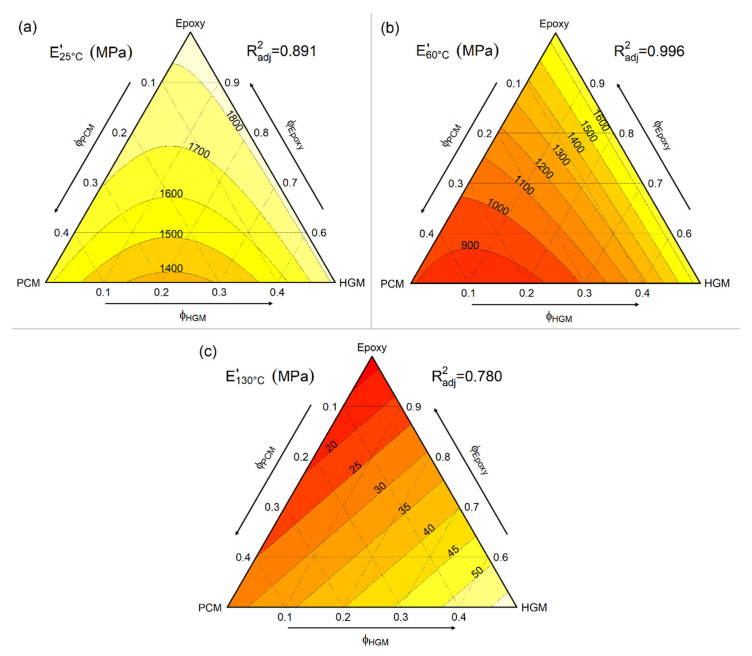
Fit-model of storage modulus (E′) values at (**a**) 25 °C, (**b**) at 60 °C, and (**c**) at 130 °C from DMTA tests on the prepared syntactic foams.

Figure 8a,b and Figure 9a–c summarizes DMTA results. Figure 8a,b demonstrates the trend of the storage modulus (E′) and the loss tangent (tanδ) of the prepared foams as a function of temperature. For neat epoxy, E′ decreases with temperature in the entire investigated temperature range but the decrease is faster at approximately 90–100 °C as the sample undergoes glass transition. This decreasing step in E′ is paralleled by a peak in tanδ. The introduction of HGM does not substantially modify the DMTA behavior of the epoxy matrix since the samples EPG-0.30 and EPG-0.40 also show a single relaxation event at 90–100 °C. However, the values of E′ below the glass transition slightly decreases by increasing the HGM fraction, while the opposite is true when above 70 °C. Moreover, the E′ inflection point and tanδ peak temperature are slightly shifted to higher temperatures upon HGM addition, which suggests that HGM may restrict the mobility of the polymer chains, thereby delaying the glass transition. HGM also decreases the height of the tanδ peak. These effects are similar to those reported in the literature for other epoxy/HGM systems [50,51] and it can be concluded that the effect of HGM on the viscoelastic behavior of the epoxy resin is relatively modest.

What is considerably more evident is the effect played by the PCM. The samples containing PCM show an additional transition in the interval 20–60°C with a relative maximum at approximately 40 °C related to the PCM melting. This transition is highlighted by a marked decrease in E′ and a small but evident tanδ peak, which is similar to that observed in previous works on composites containing the same PCM [21,38]. Moreover, the T_g_ of the epoxy resin is only slightly influenced by the composition and does not show a clear trend with PCM fraction, which is in agreement with DSC results. This confirms that the PCM and HGM do not interfere with the crosslinking process of epoxy and that the addition of a PCM in a microencapsulated form is suitable to preserve the thermal properties of the resin. In fact, the literature reports some examples in which an epoxy matrix was filled with other types of PCMs, namely shape-stabilized paraffins, and in those cases the PCM domains did influence the mobility of the matrix chains and the value of T_g_ [52,53].

The effect of PCM and HGM on the values of E′ is better illustrated in Figure 9a–c, which shows the fitted models of E′ at 25 °C, 60 °C, and 130 °C. At 25 °C (Figure 9a), E′ decreases by increasing filler concentration and is more influenced by PCM than by HGM. However, the effect of PCM is considerably more evident at 60 °C (Figure 9b) between the T_m_ of the PCM and the T_g_ of the epoxy matrix. Here, E′ decreases markedly by increasing the PCM fraction, as the PCM is melted, but it is nearly constant by varying only the HGM content. On the other hand, above the epoxy’s T_g_ (130 °C, Figure 9c), the filler content has an opposite effect on E′. In fact, E′ increases with the filler content and especially with the HGM concentration since these stiff particles help in retaining some mechanical properties that are also above the T_g_ of the matrix.

Although both PCM and HGM generally decreases the value of E′, it is important to assess whether this decrease is only due to a decrease in density. In fact, parameters such as the stiffness and strength normalized by density give a more accurate evaluation of the structural performance of the materials than absolute stiffness and strength; thus, they are more often employed for structural design [54]. The resulting specific storage moduli (E′/ρ) (Table 3) evidences the positive contribution of HGM at all the investigated temperatures and this effect increases with temperature. For instance, the sample EPG-0.40 shows an E′/ρ value of 2444 MPa/(g/cm^3^) at 25 °C (+48 % than neat epoxy), of 2256 MPa/(g/cm^3^) at 60 °C (+54%), and of 77 MPa/(g/cm^3^) at 130 °C (+600%).

The contribution of the PCM is even more temperature-dependent. In fact, at 25 °C the PCM slightly increases the value of E′/ρ since the sample EPG-40.0 shows an E′/ρ of 1680 MPa/(g/cm^3^) (+1.5% than neat epoxy). On the other hand, PCM strongly decreases E′/ρ at 60 °C since the paraffinic core is completely melted but the epoxy matrix is still in the glassy state. For example, the E′/ρ value of EPG-40.0 foam is 925 MPa/(g/cm^3^) (−37 % than neat epoxy). Finally, at 130 °C the PCM increases E′/ρ up to 29 MPa/(g/cm^3^) for the sample EPG-40.0 (+163 % than neat epoxy).

In conclusion, even though none of the fillers raise E′, the reduction in density given by the combination of PCM and HGM leads to a significant increase in E′/ρ, especially at elevated temperatures. Such materials could therefore be applied as lightweight materials with good specific stiffness in various structural applications.

**Figure 10:**
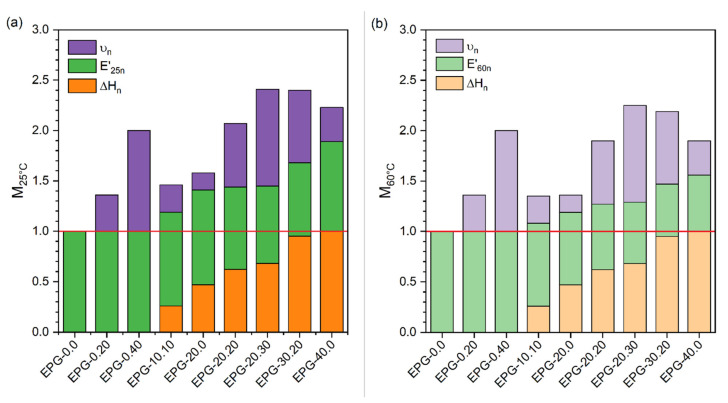
Bar charts representing the multifunctional factor M at (**a**) 25 °C (M_25°C_) and (**b**) 60 °C (M_60°C_).

The results presented so far highlight that the thermal and mechanical properties of these ternary syntactic foams strongly depend on the relative amount of HGM and PCM. In order to select the most suitable composition that optimally combines low density, high mechanical properties, and TES capability a parameter M representing the multifunctional efficiency was calculated as reported in Equation (4):

(4) $$M={\Delta H}_{n}+{E\prime }_{n}+\upsilon_{n}$$

where ΔH_n_, E′_n_, and υ_n_ are the normalized enthalpy, the normalized E′, and the normalized specific volume, respectively. These three parameters have been calculated by Equations (5)–(7):

(5) $$\Delta H_{n}=\Delta H_{m1}/max\left(\Delta H_{m1}\right)$$

(6) $$E\prime_{n}=E\prime /max\left(E\prime \right)$$

(7) $$\upsilon_{n}=\frac{\frac{\upsilon }{\min\left(\upsilon \right)}-1}{\max\left(\frac{\upsilon }{\min\left(\upsilon \right)}-1\right)}$$

where E′ was extracted from the ternary plots at 25 °C and 60 °C (Figure 9a,b) and υ is the specific volume 1/ρ. By using the ternary plots, the M values of some untested compositions were calculated and reported. The results shown in Figure 10a,b evidences that the compositions EPG-20.20, EPG-20.30, and EPG 30.20 exhibited the highest value of M and therefore are the best combination of properties and the optimal compromise between TES performance, lightness, and stiffness. Moreover, by choosing the right combination of constituents, one can optimize just one of these parameters (ΔH_m_, E′, or ρ) according to the application requirements.

## 4. Conclusions

In this paper, the incorporation of a microencapsulated PCM into epoxy/HGM syntactic foams resulted in novel multifunctional materials combining low density and thermal management ability. Dynamic rheological tests showed that both PCM and HGM considerably increased the viscosity of the uncured mixtures at elevated filler amounts (up to +630% for EPG-40.0 at $\dot{\gamma }$ = 0.2 s^−1^) and the effect of PCM was more marked than that of HGM because of its smaller size (20 µm vs 60 µm). Moreover, systems containing both PCM and HGM always showed a lower viscosity than foams with a single filler type at the same total filler volume fraction because of the higher maximum packing factors of bimodal filler distributions. Additionally, none of the fillers significantly modified the gel time nor the activation energy of the curing process.

HGM strongly decreased the gravimetric density, which ranged from 1.137 g/cm^3^ of neat epoxy down to 0.735 g/cm^3^ of the sample EPG-0.40. Furthermore, HGM considerably decreased the thermal diffusivity and conductivity, as is evidenced by LFA, while the PCM decreased the thermal conductivity only marginally. On the other hand, PCM addition determined an increase in the TES properties. In fact, DSC evidenced an increase in melting enthalpy with the PCM content up to 68 J/g with a PCM amount of 40 vol% and temperature profiling tests highlighted interesting thermal management properties in the transient regime.

Finally, DMTA tests showed that E′ was generally decreased by both HGM and PCM additions below the T_g_ of the epoxy matrix, while above T_g_ the presence of stiff particles (especially HGM) promoted an increase in E′ when compared to unfilled epoxy. On the other hand, the values of specific storage modulus (E′/ρ) increased with the HGM concentration and this was more evident at higher temperatures. The PCM generally decreased E′/ρ, especially below the T_g_ of the epoxy matrix.

Overall, these results showed that the main asset of these ternary multifunctional systems was their versatility since the property set could be tuned according to the requirements of specific applications. The selection of the best composition could be facilitated by the proposed linear ternary fitting models, which further allowed the evaluating of properties of intermediate compositions between those experimentally investigated in this work. For instance, some compositions showing balanced properties and the highest values of the multifunctional parameter M could be EPG-20.20 and EPG-30.20. In fact, EPG-20.20 had a density of 0.872 g/cm^3^, a heat storage capacity of 45 J/g, and a thermal conductivity of 0.17 W/(m∙K) while EPG-30.20 showed a predicted density of 0.85 g/cm^3^, heat storage capacity of 68 J/g, and thermal conductivity of 0.16–0.17 W/(m∙K).

The presented results can be considered as a general and useful guide to develop epoxy/HGM syntactic foams containing microencapsulated PCMs, which is promising in applications where highly specific mechanical properties, low thermal conductivity, and thermal management properties are required simultaneously, such as the transportation and refrigeration industries.

## Figures and Tables

**Table 1:** List of the prepared samples with their nominal composition.

Sample	Epoxy (vol%)/(wt%)	PCM (Vol%)/(wt%)	HGM (vol%)/(wt%)
** EPG-0.0 **	100.0/100.0	0.0/0.0	0.0/0.0
** EPG-0.10 **	90.0/98.5	0.0/0.0	10.0/1.5
** EPG-0.20 **	80.0/96.7	0.0/0.0	20.0/3.3
** EPG-0.30 **	70.0/94.5	0.0/0.0	30.0/5.5
** EPG-0.40 **	60.0/91.6	0.0/0.0	40.0/8.4
** EPG-10.0 **	90.0/91.6	10.0/8.4	0.0/0.0
** EPG-10.10 **	80.0/89.3	10.0/9.2	10.0/1.5
** EPG-10.20 **	70.0/86.5	10.0/10.1	20.0/3.4
** EPG-10.30 **	60.0/83.0	10.0/11.3	30.0/5.7
** EPG-20.0 **	80.0/83.0	20.0/17.0	0.0/0.0
** EPG-20.10 **	70.0/79.7	20.0/18.7	10.0/1.6
** EPG-20.20 **	60.0/75.8	20.0/20.7	20.0/3.5
** EPG-30.0 **	70.0/74.0	30.0/26.0	0.0/0.0
** EPG-30.10 **	60.0/69.8	30.0/28.6	10.0/1.6
** EPG-40.0 **	60.0/64.6	40.0/35.4	0.0/0.0

**Table 2:** Main results of DSC tests on the prepared foams (first and second scans).

Sample	c_p30_ J∙g^−1^K^−1^	T_m1_ °C	ΔH_m1_ J∙g^−1^	T_g1_ °C	T_c_ °C	ΔH_c_ J∙g^−1^
**EPG-0.0**	1.34	-	0.0	91.8	-	0.0
**EPG-0.10**	1.36	-	0.0	92.1	-	0.0
**EPG-0.20**	1.29	-	0.0	89.2	-	0.0
**EPG-0.30**	1.34	-	0.0	89.3	-	0.0
**EPG-0.40**	1.28	-	0.0	85.3	-	0.0
**EPG-10.0**	1.45	48.2	16.5	91.6	22.7	16.5
**EPG-10.10**	1.47	49.1	17.8	92.8	22.0	16.2
**EPG-10.20**	1.40	47.6	18.4	87.9	23.5	17.6
**EPG-10.30**	1.39	47.8	24.2	91.1	23.2	21.4
**EPG-20.0**	1.46	50.5	36.3	88.3	21.4	34.7
**EPG-20.10**	1.48	52.7	46.6	89.0	19.7	41.8
**EPG-20.20**	1.45	50.4	44.5	89.4	21.2	42.1
**EPG-30.0**	1.47	51.2	55.6	89.1	20.8	56.5
**EPG-30.10**	1.53	52.0	60.4	85.9	19.7	58.5
**EPG-40.0**	1.53	53.0	67.6	89.3	19.3	69.7
**PCM**	1.86	45.9	218.1	-	27.9	217.0

c_p30_ = specific heat at 30 °C; T_m1_ = melting temperature of the PCM; ΔH_m1_ = melting enthalpy of the PCM; T_g1_ = glass transition temperature of the epoxy matrix in the first heating scan; T_c_ = crystallization temperature of the PCM; ΔH_c_ = crystallization enthalpy of the PCM.

**Table 3:** Selected DMTA data and specific storage modulus E′/ρ at different temperatures of the prepared foams.

Sample	E″ Peak °C/MPa	tanδ Peak °C/-	E′/ρ MPa/(g/cm^3^) T = 25 °C	E′/ρ MPa/(g/cm^3^) T = 60 °C	E′/ρ MPa/(g/cm^3^) T = 130 °C
**EPG-0.0**	94/201	105/0.85	1655	1469	11
**EPG-0.10**	98/198	106/0.63	1646	1499	24
**EPG-0.30**	99/207	108/0.64	2073	1886	34
**EPG-0.40**	100/204	108/0.56	2444	2256	77
**EPG-10.30**	99/160	107/0.57	1982	1551	51
**EPG-30.0**	96/124	107/0.60	1642	100	20
**EPG-30.10**	88/118	98/0.52	1618	1035	28
**EPG-40.0**	94/114	102/0.53	1680	925	29

## Data Availability

The data presented in this study are available upon request from the corresponding author.

## Abbreviations

α	thermal diffusivity of the samples at 30 °C measured by LFA	mm^2^/s
$\dot{\gamma }$	shear rate	s^−1^
$\Delta H_{m}$	specific enthalpy of fusion	J/g
$\Delta H_{c}$	specific enthalpy of crystallization	J/g
η	viscosity	Pa∙s
$\lambda_{30{}^{\circ}C}$	thermal conductivity at 30 °C	W/(m∙K)
υ	specific volume (i.e., 1/ρ)	cm^3^/g
ρ	pyconmeter density at 23 °C	g/cm^3^
$\phi_{Epoxy}$	volume fraction of epoxy	-
$\phi_{PCM}$	volume fraction of PCM	-
$\phi_{HGM}$	volume fraction of HGM	-
c_p_	specific heat capacity	J/(g∙K)
E′	storage modulus	MPa
E″	loss modulus	MPa
G′	shear storage modulus	MPa
G″	shear loss modulus	MPa
M	multifunctional efficiency coefficient	-
$t_{26-55}$	time for heating from 26 °C to 55 °C	min
$t_{55-26}$	time for cooling from 55 °C to 26 °C	min
tanδ	loss tangent (i.e., E′’/E′)	-
T_c_	crystallization temperature	°C
T_g_	glass transition temperature	°C
T_m_	melting temperature	°C
